# Solving ordinary and partial differential equations using an analog computing system based on ultrasonic metasurfaces

**DOI:** 10.1038/s41598-023-38718-1

**Published:** 2023-08-18

**Authors:** Robert Frederik Uy, Viet Phuong Bui

**Affiliations:** 1 Hwa Chong Institution, 661 Bukit Timah Road, Singapore, 269734 Singapore; 2 Institute of High Performance Computing (IHPC), Agency for Science, Technology and Research (A*STAR), 1 Fusionopolis Way, #16-16 Connexis, Singapore, 138632 Republic of Singapore

**Keywords:** Applied physics, Acoustics, Physics, Mathematics and computing, Applied mathematics

## Abstract

Wave-based analog computing has recently emerged as a promising computing paradigm due to its potential for high computational efficiency and minimal crosstalk. Although low-frequency acoustic analog computing systems exist, their bulky size makes it difficult to integrate them into chips that are compatible with complementary metal-oxide semiconductors (CMOS). This research paper addresses this issue by introducing a compact analog computing system (ACS) that leverages the interactions between ultrasonic waves and metasurfaces to solve ordinary and partial differential equations. The results of our wave propagation simulations, conducted using MATLAB, demonstrate the high accuracy of the ACS in solving such differential equations. Our proposed device has the potential to enhance the prospects of wave-based analog computing systems as the supercomputers of tomorrow.

## Introduction

A plethora of electronic and mechanical analog computers have been developed in the past two millennia to solve mathematical equations and perform mathematical operations with increased efficiency^1–3^, but they were later replaced by more advanced digital computers^2,3^. In view of the recent advancements in the field of metamaterials, interest in analog computing has been revived, with the focus being on wave-based analog computing^1,3,8^. These new computing systems leverage the properties of waves and metasurfaces to solve mathematical equations and perform mathematical operations to satisfy the need for ever-greater computational efficiency and capacity^6,7^ amidst the grim outlook for further augmentation of digital computers as Moore’s law approaches its physical limitations^2,4,5^.

Due to their powerful parallel processing, high computational efficiency, and minimal crosstalk, wave-based analog computing systems have been hailed as a potential future of computing^1,8,9^. It was the pioneering work of Silva et al.^10^ on computational metamaterials that set the stage for subsequent research into analog computing systems that perform mathematical operations and solve equations^1–4,6–35,37^, with a subset of these focusing on the use of the Fourier transform (FT) to do so^3,6,9,10,26–28,37^. More recently, Zangeneh-Nejad et al. provided a well-written, comprehensive overview of recent developments in this field as a whole^1^.

In the realm of acoustics, Zuo et al. designed and tested an acoustic analog computing system based on labyrinthine metasurfaces to solve $n$ ^th^-order inhomogeneous ordinary differential equations^9^. Many other studies on acoustic analog computing systems have also been carried out, but all such systems operate in the kilohertz (kHz) frequency range^3,9,26–28^. Even when thin planar metasurfaces are used, a physically bulky computing system is required for analog computing at such low frequencies (long wavelengths). In this paper, we propose a solution to this problem: a compact ultrasonic analog computing system (ACS) with a working frequency in the gigahertz (GHz) range. Due to the relatively shorter wavelength of GHz ultrasonic waves, our proposed ACS is far less bulky and can consequently be easily integrated into CMOS-compatible chips.

This paper is organized as follows. Firstly, we present the ACS’ architecture and elaborate on its working principle. Next, the ability of the ACS to solve differential equations is demonstrated, including a comprehensive error and accuracy optimization analysis for each type of differential equation and each type of function. Following this, we discuss our study’s key findings and conclusions, relating them to the wider context of wave-based analog computing. Finally, we provide a comprehensive account of our research methodology—including information on the design process for the ultrasonic metalens, the simulation of wave propagation through the ACS, and important considerations for the selection of simulation parameters.

## Architecture and working principle of the analog computing system (ACS)

### Architecture

**Figure 1:**
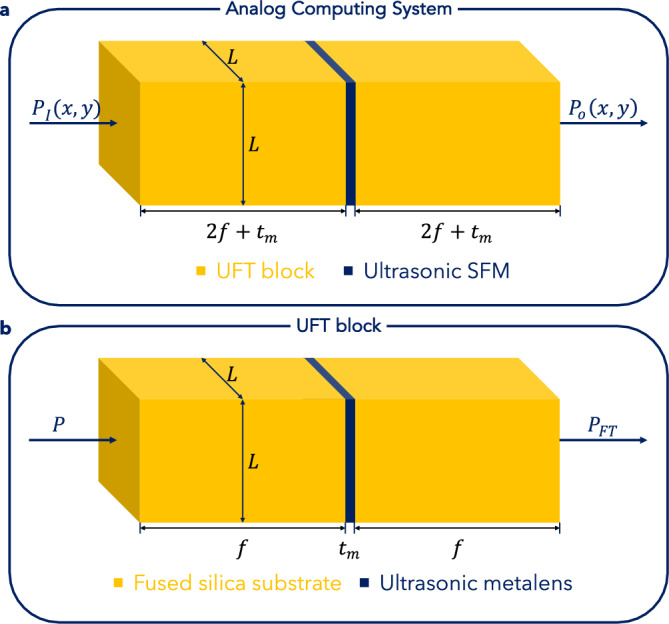
Schematic of the ACS and the UFT block. (**a**) The figure features a schematic of the proposed ACS, which consists of three main parts: a UFT block, an SFM, and another UFT block. (**b**) The figure features a schematic of the UFT block, which has three key components: a substrate layer, the ultrasonic metalens, and another substrate layer.

Our proposed ACS (Fig. 1a) is made up of three key components: an Ultrasonic Fourier Transform (UFT) block, a spatial filtering metasurface (SFM), and another UFT block. The pressure fields at the input and output planes of the ACS are $P_{I} \left( {x,y} \right)$ and $P_{O} \left( {x,y} \right)$, respectively. The side length of the entire ACS’ square cross-section is $L$.

Referring to Fig. 1b, each UFT block consists of three main parts: a fused silica substrate layer, the ultrasonic metalens, followed by another fused silica substrate layer. If the input pressure field of the ultrasonic wave that is made to pass through the UFT block is $P$, the output pressure field obtained at the other end of the UFT block is $P_{FT}$, which has been shown to be proportional to $P$’s Fourier transform^37^. A key condition for obtaining the UFT through this block is that both the thickness of the substrate layers and the focal length of the metalens must be $f$ ^37^. The metalens’ thickness is $t_{m}$, as shown in Fig. 1b.

Our proposed ACS is designed to operate at a frequency of $f_{wave} = 1.7$ GHz, which is a high ultrasonic frequency. This enables greater compactness, making it easier to integrate the ACS into CMOS-compatible chips. Each substrate layer has a thickness of $f = 1.0886$ mm and is made of fused silica (which we chose for its isotropy as a material). In fused silica, the speed of ultrasonic waves is $v_{wave} = 5880$ m s^-1^, from which we can calculate the wavelength to be $\lambda = v_{wave} /f_{wave} = 3.46$ µm.

**Figure 2:**
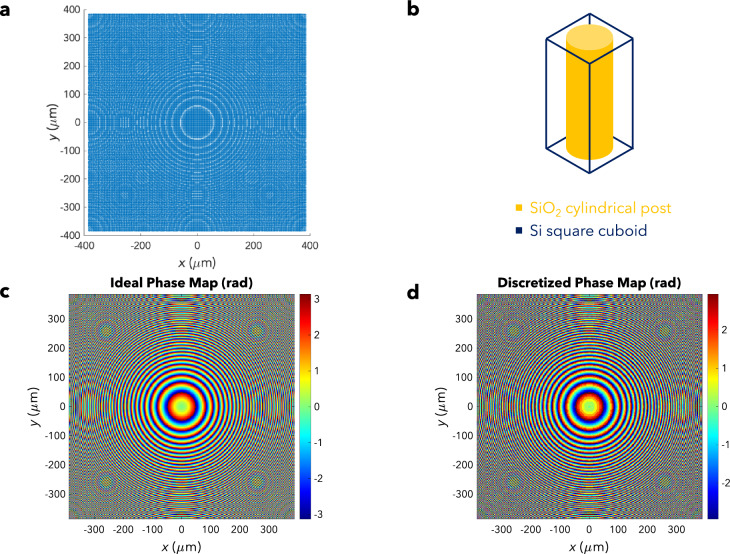
Ultrasonic Metalens. (**a**) Ultrasonic metalens – top view. (**b**) Unit cell – a SiO_2_ cylindrical post (gold) embedded in a Si square cuboid (dark blue). (**c**) Ideal Phase Map. (**d**) Discretized Phase Map. Adapted from Ref. 37.

In Fig. 2a, the metalens is made up of several unit cells, each of which has a thickness of $t_{m} = 16$ µm and a square cross-section of side length 3 µm (a subwavelength feature). Each unit cell (Fig. 2b) is composed of a square cuboid made of Si with a cylindrical post made of SiO_2_ embedded in it. According to the theoretical working principle of the ACS, the ultrasonic metalens ought to obey a paraboloidal phase profile

1 $$\begin{array}{*{20}c} {\phi_{ideal} \left( {x,y} \right) = k\left( {\frac{{x^{2} + y^{2} }}{2f}} \right)} \\ \end{array}$$

such that the pressure field $P_{FT}$ would be proportional to the FT of $P$. Due to the limited number of distinct unit cells available, however, discretization is required. Therefore, the cylindrical post radius of each unit cell must correspond to the interpolated phase shift at that point (after discretization). The process of interpolation transforms the ideal phase map (Fig. 2c) into the discretized phase map (Fig. 2d) that is later used for phase-to-radius mapping.

In addition, the transmission coefficient function $T\left( {x,y} \right)$ of the SFM must correspond to the transfer function (TF) $H\left( {k_{x} ,k_{y} } \right)$ required to solve a particular ordinary or partial differential equation.

### Working principle

Uy and Bui^37^ have previously determined that the input $P$ and the output $P_{FT}$ of the UFT block are approximately (see Table 1 for list of approximations) related by

2 $$\begin{array}{*{20}c} {P_{FT} \left( {u,v} \right) = \frac{{j\exp \left( { - 2jkf} \right)}}{\lambda f}{\mathcal{F}}\left\{ {P\left( {\xi ,\eta } \right)} \right\},} \\ \end{array}$$

where $j$ is the imaginary unit, $\lambda$ is the wavelength, $k$ is the wavenumber, and the operator ${\mathcal{F}}$ denotes the FT.

**Table 1:** List of approximations required to achieve $P_{FT} \propto {\mathcal{F}}\left\{ P \right\}$.

Approximation	Validity
$\left| {{\varvec{r}} - {\varvec{r}}_{0} } \right| \approx f\left[ {1 + \frac{1}{2}\left( {\frac{x - \xi }{f}} \right)^{2} + \frac{1}{2}\left( {\frac{y - \eta }{f}} \right)^{2} } \right]$	Fresnel
$\left| {{\varvec{r}} - {\varvec{r}}_{0} } \right| \approx f$	Paraxial
$\cos \left( {{\varvec{n}},{\varvec{r}} - {\varvec{r}}_{0} } \right) \approx 1$	Paraxial
$\frac{1}{{\left| {{\varvec{r}} - {\varvec{r}}_{0} } \right|}} + jk \approx jk$	Distances much larger than $\lambda$

Now, let $f\left( {x,y} \right)$ and $g\left( {x,y} \right)$ be the input and output, respectively, of a particular ODE or PDE. It can be shown that $f\left( {x,y} \right)$ and $g\left( {x,y} \right)$ are mathematically related by the equation

3 $$\begin{array}{*{20}c} {g\left( {x,y} \right) = {\mathcal{F}}^{ - 1} \left\{ {H\left( {k_{x} ,k_{y} } \right){\mathcal{F}}\left\{ {f\left( {x,y} \right)} \right\}} \right\},} \\ \end{array}$$

where the operator ${\mathcal{F}}^{ - 1}$ denotes the inverse FT and $H\left( {k_{x} ,k_{y} } \right)$ is the transfer function for a certain ODE or PDE.

At first glance, Eqs. (2) and (3) might seem to suggest that the ACS cannot compute the accurate result. For one, the UFT block yields an output $P_{FT}$ that is only proportional to—but not actually equal to—the FT of the input $P$. Moreover, it is essential to note that the correct output is obtained by taking the inverse FT of $H\left( {k_{x} ,k_{y} } \right){\mathcal{F}}\left\{ {f\left( {x,y} \right)} \right\}$, whereas the second UFT block calculates the FT (not the inverse FT). However, these concerns do not actually hinder the ACS from yielding the desired output. In fact, the mirror image of the correct output $g\left( {x,y} \right)$ is given by the equation

4 $$\begin{array}{*{20}c} {g\left( { - x, - y} \right) = \frac{1}{{\left( {\lambda f} \right)^{2} }}{\mathcal{F}}\left\{ {H\left( {k_{x} ,k_{y} } \right){\mathcal{F}}\left\{ {f\left( {x,y} \right)} \right\}} \right\},} \\ \end{array}$$

which is the additive inverse of the magnitude of the ACS’ output $P_{O} \left( {x,y} \right)$. Therefore, to achieve the desired result $g\left( {x,y} \right)$, we simply need to take the mirror image of the ACS’ output $P_{O} \left( {x,y} \right)$ and subsequently obtain its additive inverse.

It is also important to recognize that the transmission coefficient can only have amplitudes of less than or equal to 1. If this condition is satisfied by $H\left( {k_{x} ,k_{y} } \right)$, then all is well, and $H\left( {k_{x} ,k_{y} } \right)$ would also be the transmission coefficient function. However, this is not necessarily satisfied by all transfer functions. In such cases, we would have to normalize the transfer function such that it satisfies this condition. Consequently, we would also not obtain the exact output $g\left( {x,y} \right)$ but rather a scaled version of it. Nevertheless, we can recover the desired result by appropriately rescaling the output.

## Results

A function that is space-limited has non-zero magnitude values only within in a finite region in the space domain, whereas a function that is bandlimited is one that has a finite spectral width that includes all spatial frequency components with non-zero magnitude values. It should be noted that it is not possible for a function to be both space-limited and bandlimited^38^. Furthermore, functions that are space-limited but not bandlimited, such as the rect function, are not of particular interest in the context of solving differential equations. In this paper, we thus consider two main kinds of functions: (1) bandlimited but not space-limited and (2) neither space-limited nor bandlimited.

In the wave propagation simulations, we used the Sinc and Gaussian functions as archetypal examples for each kind. The Sinc functions follow the general form $f\left( x \right) = {\text{sinc}}\left( {x/w} \right)$, where the parameter $w \in {\mathbb{R}}^{ + }$ is an indication of the Sinc function’s geometric spread. The Gaussian functions, on the other hand, take the form $f\left( x \right) = \exp \left( { - \pi x^{2} /\gamma^{2} } \right)$, where the parameter $\gamma \in {\mathbb{R}}^{ + }$ serves as a measure of the Gaussian function’s geometric spread. Additionally, we chose the root-mean-squared error (RMSE) after normalization as the error metric used to assess the accuracy of the ACS’ output, relative to the analytical solution.

### Ordinary differential equations (ODE)

#### Mathematical basis

Generalizing the derivative property of the FT^39^,

5 $$\begin{array}{*{20}c} {{\mathcal{F}}\left\{ {\frac{{d^{n} f\left( x \right)}}{{dx^{n} }}} \right\} = \left( {jk_{x} } \right)^{n} {\mathcal{F}}\left\{ {f\left( x \right)} \right\}.} \\ \end{array}$$

Consider a general $n$ ^th^-order inhomogeneous ODE

6 $$\begin{array}{*{20}c} {f\left( x \right) = c_{n} g^{\left( n \right)} \left( x \right) + c_{n - 1} g^{{\left( {n - 1} \right)}} \left( x \right) + \ldots + c_{1} g^{\prime}\left( x \right) + c_{0} g\left( x \right).} \\ \end{array}$$

Taking the FT of both sides of Eq. (6), we obtain

7 $$\begin{array}{*{20}c} {{\mathcal{F}}\left\{ {f\left( x \right)} \right\} = {\mathcal{F}}\left\{ {g\left( x \right)} \right\}\mathop \sum \limits_{i = 0}^{n} c_{i} \left( {jk_{x} } \right)^{i} ,} \\ \end{array}$$

from which we can deduce that

8 $$\begin{array}{*{20}c} {g\left( x \right) = {\mathcal{F}}^{ - 1} \left\{ {\left[ {\mathop \sum \limits_{i = 0}^{n} c_{i} \left( {jk_{x} } \right)^{i} } \right]^{ - 1} {\mathcal{F}}\left\{ {f\left( x \right)} \right\}} \right\}.} \\ \end{array}$$

Note that Eq. (8) can be re-expressed as

9 $$\begin{array}{*{20}c} {g\left( x \right) = \frac{1}{{4\pi^{2} }}\mathop {\iint }\limits_{ - \infty }^{\infty } \left[ {\mathop \sum \limits_{i = 0}^{n} c_{i} \left( {jk_{x} } \right)^{i} } \right]^{ - 1} {\mathcal{F}}\left\{ {f\left( x \right)} \right\}\exp \left[ {j\left( {k_{x} x + k_{y} y} \right)} \right]dk_{x} dk_{y} .} \\ \end{array}$$

By definition, the spatial frequencies are $k_{x} = - 2\pi x/\lambda f$ and $k_{y} = - 2\pi y/\lambda f$, so we have $dk_{x} = - \left( {2\pi /\lambda f} \right)dx$ and $dk_{y} = - \left( {2\pi /\lambda f} \right)dy$. Therefore, using these substitutions and then replacing $x$ with $- x$ and $y$ with $- y$,

10 $$\begin{array}{*{20}c} {g\left( { - x} \right) = \frac{1}{{\left( {\lambda f} \right)^{2} }}\mathop {\iint }\limits_{ - \infty }^{\infty } \left[ {\mathop \sum \limits_{i = 0}^{n} c_{i} \left( {jk_{x} } \right)^{i} } \right]^{ - 1} {\mathcal{F}}\left\{ {f\left( x \right)} \right\}\exp \left[ { - j\left( {k_{x} x + k_{y} y} \right)} \right]dxdy = \frac{1}{{\left( {\lambda f} \right)^{2} }}{\mathcal{F}}\left\{ {\left[ {\mathop \sum \limits_{i = 0}^{n} c_{i} \left( {jk_{x} } \right)^{i} } \right]^{ - 1} {\mathcal{F}}\left\{ {f\left( x \right)} \right\}} \right\},} \\ \end{array}$$

It is apparent from Eq. (10) that $f\left( x \right)$ is the input and

11 $$\begin{array}{*{20}c} {H\left( {k_{x} } \right) = \left[ {\mathop \sum \limits_{i = 0}^{n} c_{i} \left( {jk_{x} } \right)^{i} } \right]^{ - 1} } \\ \end{array}$$

is the transfer function needed to solve *n*th-order inhomogeneous ODEs of the form given in Eq. (6)^9^.

#### Simulation results

In the simulations, we used the ODE

12 $$\begin{array}{*{20}c} {4g^{{\prime \prime }} \left( x \right) - 8g^{{\prime }} \left( x \right) + 16g\left( x \right) = f\left( x \right).} \\ \end{array}$$

**Figure 3:**
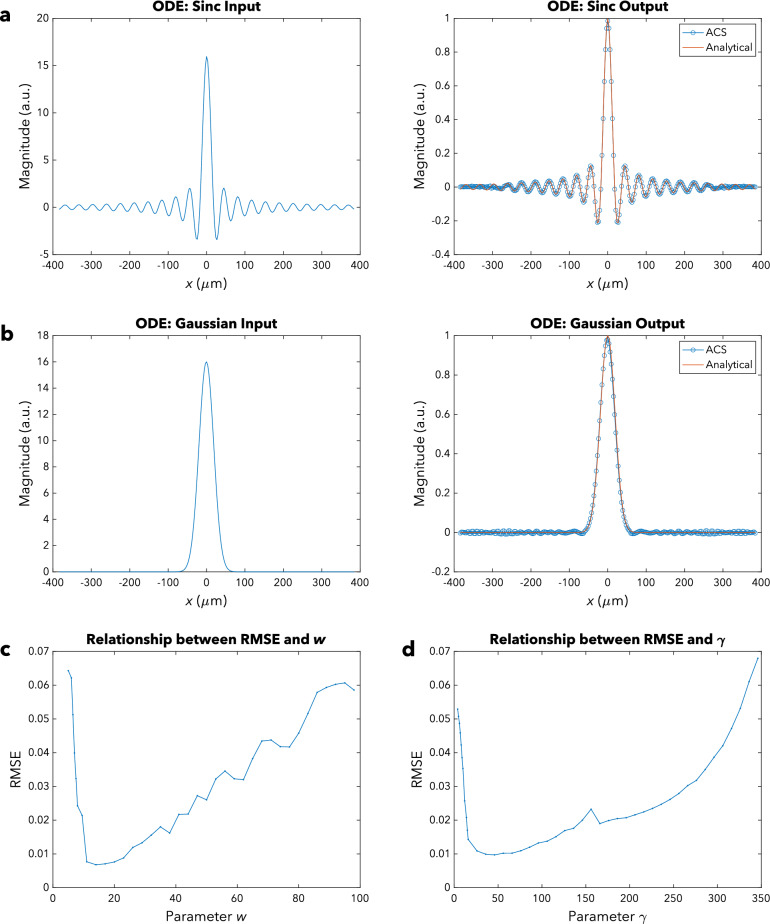
Ordinary Differential Equation (ODE) Simulations. (**a**) Sinc function: Input (left) and output (right) magnitude profiles for $w = 18$. The blue line with circled data points indicates the simulated output of the ACS, whereas the orange line shows the analytical solution. (**b**) Gaussian function: Input (left) and output (right) magnitude profiles for $\gamma = 48$. The blue line with circled data points indicates the simulated output of the ACS, whereas the orange line shows the analytical solution. (**c**) Relationship between the RMSE and the parameter $w$ for the Sinc function. (**d**) Relationship between the RMSE and the parameter $\gamma$ for the Gaussian function.

For the first type of function, the solution $g\left( x \right)$ is a Sinc function with parameter $w = 18$. The RMSE after normalization, based on our simulation, was 0.00737. From Fig. 3a, we can observe that the analytical solution and the ACS’ output are in excellent agreement, especially near the center. There are some minor discrepancies towards the edges, but these are actually expected since we know that the FT is only obtained in the paraxial region (see approximations listed in Table 1). Furthermore, metalens aberration—as a result of discretization of the metalens’ phase profile—also contributes, albeit very minimally, to the observed deviations. It should also be noted that there is some undersampling due to truncation of the input function $f\left( x \right)$ as well as aliasing arising from bandlimiting of the output function $g\left( x \right)$.

The simulation for the second type of function was conducted with a Gaussian function with parameter $\gamma = 48$ as the solution $g\left( x \right)$. In this case, the RMSE after normalization was found to be 0.00975. Figure 3b shows that the output of the ACS and the analytical solution agree very well with each other. Granted, there are small lobes towards the edges, largely due to metalens aberration as well as the paraxial approximation requirement not being met. There are also minor errors associated with undersampling of the input and bandlimiting of the output. Be that as it may, the output nonetheless matches the analytical solution very well.

#### Optimization of accuracy

Figures 3c and d show the relationship between the RMSE and the geometric spread parameters $w$ and $\gamma$, respectively, for solving ODEs. We can observe that the RMSE initially decreases then increases as $w$ or $\gamma$ is increased. This two-part trend in the RMSE is mainly caused by two factors: (1) the level of aliasing and undersampling and (2) the validity of the paraxial approximations.

Firstly, as $w$ or $\gamma$ is initially increased, there is reduced aliasing in the output plane of the first UFT block and reduced undersampling in the input plane of the second UFT block, resulting in a fall in the RMSE. However, as $w$ or $\gamma$ continues to increase past a certain threshold, there is increased undersampling in the input plane of the first UFT block and increased aliasing in the output plane of the second UFT block that drive the observed rise in the RMSE.

Secondly, the paraxial approximations initially become more valid as $w$ or $\gamma$ increases, then it becomes less valid as $w$ or $\gamma$ increases further. As $w$ or $\gamma$ initially increases, the energy becomes less highly concentrated near the center of the first UFT block’s input plane and less extensively spread out in the first UFT block’s output plane. The energy also becomes less extensively spread out in the second UFT block’s input plane and less highly concentrated near the center of the second UFT block’s output plane. Then, as $w$ or $\gamma$ increases further, the energy becomes more extensively spread out in the first UFT block’s input plane and more highly concentrated near the center of the first UFT block’s output plane. Moreover, the energy becomes more highly concentrated near the center of the second UFT block’s input plane and more extensively spread out in the second UFT block’s output plane.

Therefore, to optimize accuracy, the input function $f\left( x \right)$ can be scaled parallel to $x$ such that the geometric spread parameter $w$ or $\gamma$ takes on a moderate value. The output $P_{O} \left( {x,y} \right)$ of the ACS can then be appropriately rescaled back to obtain the desired solution $g\left( x \right)$.

Refer to Supplementary Figs. S2 to S5 for additional diagrams in support of the above explanation.

### Partial differential equations (PDE)

#### Mathematical basis

Consider the partial differential equation

13 $$\begin{array}{*{20}c} {\kappa_{1} \nabla^{2} g\left( {x,y} \right) + \kappa_{2} g\left( {x,y} \right) = f\left( {x,y} \right).} \\ \end{array}$$

Taking the FT of both sides of Eq. (13),

14 $$\begin{array}{*{20}c} {{\mathcal{F}}\left\{ {f\left( {x,y} \right)} \right\} = \left[ { - \kappa_{1} \left( {k_{x}^{2} + k_{y}^{2} } \right) + \kappa_{2} } \right]{\mathcal{F}}\left\{ {g\left( {x,y} \right)} \right\},} \\ \end{array}$$

which can be re-arranged to get

16 $$\begin{array}{*{20}c} {g\left( {x,y} \right) = {\mathcal{F}}^{ - 1} \left\{ {\frac{{{\mathcal{F}}\left\{ {f\left( {x,y} \right)} \right\}}}{{ - \kappa_{1} \left( {k_{x}^{2} + k_{y}^{2} } \right) + \kappa_{2} }}} \right\}.} \\ \end{array}$$

In its integral form, Eq. (15) can be re-written as

16 $$\begin{array}{*{20}c} {g\left( {x,y} \right) = \frac{1}{{4\pi^{2} }}\mathop {\iint }\limits_{ - \infty }^{\infty } \frac{{{\mathcal{F}}\left\{ {f\left( {x,y} \right)} \right\}}}{{ - \kappa_{1} \left( {k_{x}^{2} + k_{y}^{2} } \right) + \kappa_{2} }}\exp \left[ {j\left( {k_{x} x + k_{y} y} \right)} \right]dk_{x} dk_{y} .} \\ \end{array}$$

Substituting $dk_{x} = - \left( {2\pi /\lambda f} \right)dx$ and $dk_{y} = - \left( {2\pi /\lambda f} \right)dy$ and replacing $x$ with $- x$ and $y$ with $- y$ then yield

17 $$\begin{array}{*{20}c} {g\left( { - x, - y} \right) = \frac{1}{{\left( {\lambda f} \right)^{2} }}\mathop {\iint }\limits_{ - \infty }^{\infty } \frac{{{\mathcal{F}}\left\{ {f\left( {x,y} \right)} \right\}}}{{ - \kappa_{1} \left( {k_{x}^{2} + k_{y}^{2} } \right) + \kappa_{2} }}\exp \left[ { - j\left( {k_{x} x + k_{y} y} \right)} \right]dxdy = \frac{1}{{\left( {\lambda f} \right)^{2} }}{\mathcal{F}}\left\{ {\frac{{{\mathcal{F}}\left\{ {f\left( {x,y} \right)} \right\}}}{{ - \kappa_{1} \left( {k_{x}^{2} + k_{y}^{2} } \right) + \kappa_{2} }}} \right\}.} \\ \end{array}$$

Therefore, $f\left( {x,y} \right)$ is the input and

18 $$\begin{array}{*{20}c} {H\left( {k_{x} ,k_{y} } \right) = \frac{1}{{ - \kappa_{1} \left( {k_{x}^{2} + k_{y}^{2} } \right) + \kappa_{2} }}} \\ \end{array}$$

is the transfer function needed to solve PDEs of the specified form^28^.

#### Simulation results

In the simulations, we used the PDE

19 $$\begin{array}{*{20}c} {\nabla^{2} g\left( {x,y} \right) + 4g\left( {x,y} \right) = f\left( {x,y} \right).} \\ \end{array}$$

**Figure 4:**
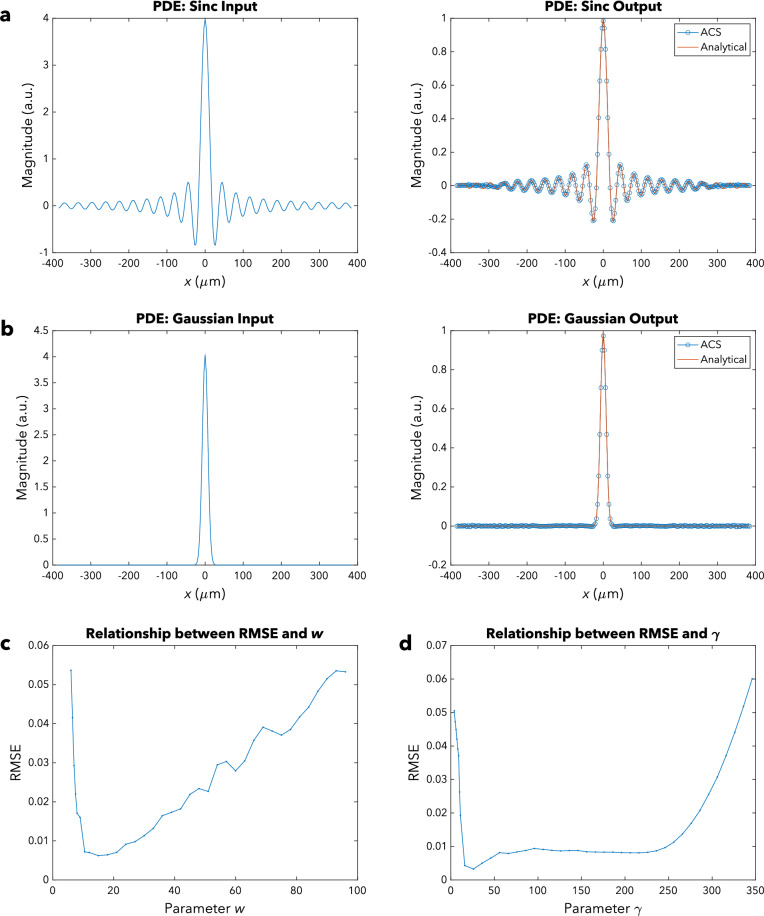
Partial Differential Equation (PDE) Simulations. (**a**) Sinc function: Input (left) and output (right) magnitude profiles for $w=18$. The blue line with circled data points indicates the simulated output of the ACS, whereas the orange line shows the analytical solution. (**b**) Gaussian function: Input (left) and output (right) magnitude profiles for $\gamma =18$. The blue line with circled data points indicates the simulated output of the ACS, whereas the orange line shows the analytical solution. (**c**) Relationship between the RMSE and the parameter $w$ for the Sinc function. (**d**) Relationship between the RMSE and the parameter $\gamma$ for the Gaussian function.

The simulation for the first type of function was carried out with a two-dimensional Sinc function with parameter $w = 18$ as the solution $g\left( x,y \right)$. The RMSE after normalization, based on our simulation, was 0.00643. We can observe from Fig. 4a that the ACS’ output and the analytical solution are in excellent agreement, particularly near the center. There are some small deviations towards the edges, which can be attributed to the paraxial approximation not being met. In addition, metalens aberration also contributes to the discrepancies. There is also some undersampling due to truncation of the input function $f\left( x,y \right)$ as well as aliasing arising from bandlimiting of the output function $g\left( x,y \right)$.

For the second type of function, the solution $g\left( x,y \right)$ is a two-dimensional Gaussian function with parameter $\gamma = 18$. In this case, the RMSE after normalization was found to be 0.00266. Figure 4b shows that the output of the ACS and the analytical solution agree very well with each other, especially in the paraxial region for the same reason cited above. Due to metalens aberration as well as the paraxial approximation requirement not being met, there are some small lobes towards the edges. What is more, undersampling of the input $f\left( x,y \right)$ and aliasing of the output function $g\left( x,y \right)$ also contribute to the observed discrepancies. Nonetheless, the output for the significant magnitude values matches the analytical solution very well.

#### Optimization of accuracy

Figure 4c and d show the relationship between the RMSE and the geometric spread parameters $w$ and $\gamma$, respectively, for solving PDEs. We can observe that as $w$ or $\gamma$ is increased, the RMSE initially falls and subsequently rises. This trend in the RMSE can be largely ascribed to two key factors: (1) the level of undersampling and aliasing and (2) the paraxial approximations’ validity.

Firstly, as $w$ or $\gamma$ is initially increased, there is less aliasing in the first UFT block’s output plane and less undersampling in the second UFT block’s input plane, so the RMSE decreases. However, as $w$ or $\gamma$ continues to increase beyond a certain value, there is increased undersampling in the first UFT block’s input plane and increased aliasing in the second UFT block’s output plane, resulting in the observed increase in the RMSE.

Secondly, the paraxial approximations initially become more valid as $w$ or $\gamma$ initially increases, then it becomes less valid as $w$ or $\gamma$ increases further. Initially, as $w$ or $\gamma$ increases, the energy becomes less highly concentrated near the center of the input plane of the first UFT block and less extensively spread out in the output plane of the first UFT block. The energy also becomes less extensively spread out in the input plane of the second UFT block and less highly concentrated near the center of the output plane of the second UFT block. Then, as $w$ or $\gamma$ increases further, the energy becomes more extensively spread out in the input plane of the first UFT block and more highly concentrated near the center of the output plane of the first UFT block. Additionally, the energy becomes more highly concentrated near the center of the input plane of the second UFT block and more extensively spread out in the output plane of the second UFT block.

Thus, accuracy can be optimized by scaling the input $f\left( x \right)$ parallel to $x$ so that the geometric spread parameter takes on a moderate value. We can then appropriately rescale back the ACS’ output $P_{O} \left( {x,y} \right)$ to obtain the desired solution.

Refer to Supplementary Figs. S6 to S9 for additional diagrams supporting the above explanation.

## Conclusion

This paper introduces an analog computing system (ACS) that uses ultrasonic waves and metasurfaces to solve ordinary and partial differential equations. Through our simulations, we have clearly demonstrated the ability of the proposed ACS to yield highly accurate results when solving both types of differential equations involving both types of functions. In contrast to other studies in existing literature, a key contribution of our paper is the exploration of how the accuracy of the ACS’ output may be optimized through the selection of an appropriate (moderate) value of the geometric spread parameter $w$ or $\gamma$.

Our study’s findings are anticipated to advance the development of wave-based analog computing systems, potentially surpassing the constraints of digital computers. This has far-reaching implications for the fields of computing and signal processing, hopefully laying the foundation for technological breakthroughs in the future.

## Methods

### Ultrasonic metalens designing process

The process of designing the ultrasonic metalens is detailed below. Refer to Supplementary Fig. S1 for a flowchart summarizing the method.

To start, it is crucial to conduct unit cell simulations in order to establish the relationship between the radius of a cylindrical post and the associated phase shift for that particular unit cell. This correlation (Phase-to-radius Mapping) serves as a reference point for subsequent steps. Next, an array of the Ideal Phase Map can be generated. It consists of phase values at sampled points following the paraboloidal phase profile

20 $$\begin{array}{*{20}c} {\phi_{ideal} \left( {x,y} \right) = k\left( {\frac{{x^{2} + y^{2} }}{2f}} \right)} \\ \end{array}$$

required to achieve $P_{FT} \propto {\mathcal{F}}\left\{ P \right\}$ theoretically^37^. The next step is to use the MATLAB function interp1 to interpolate the nearest available phase value from the unit cell simulations, and this results in the Discretized Phase Map, consisting of phase values that have a corresponding radius from the unit cell simulations. Subsequently, the phase-to-radius mapping can be used to create the Radius Map, an array of radius values at each sampled point. Finally, the MATLAB function viscircles can be used to generate a figure of the metalens, comprising unit cells with cylindrical posts whose radii correspond to the radius at that point (according to the Radius Map).

### Semi-analytical wave propagation simulations

Using the exact solutions of the Kirchhoff-Helmholtz Integral (before Fresnel and paraxial approximations as listed in Table 1 were applied), we conducted semi-analytical simulations of the propagation of ultrasonic waves through our proposed ACS. The output of each simulation was then compared with the analytical solution.

To conduct these simulations, we used MATLAB to implement the code because it is far less computationally costly as opposed to Finite Element Method (FEM) software like COMSOL Multiphysics. This is to enable us to carry out simulations involving arrays of significantly larger size—key to understanding the proposed ACS’ true capabilities.

#### Propagation within each UFT block

Through an FFT-based convolution approach, we can obtain the pressure field $\overline{P}_{M - } \left( {x,y} \right)$ at the plane before the ultrasonic metalens. This approach involves convolving the zero-padded input pressure field array $\overline{P}_{S} \left( {\xi ,\eta } \right)$ with the convolution kernel

21 $$\begin{array}{*{20}c} {\overline{h}_{1} \left( {\xi ,\eta } \right) = \frac{{j\exp \left( { - jk\sqrt {f^{2} + \xi^{2} + \eta^{2} } } \right)}}{{\lambda \sqrt {f^{2} + \xi^{2} + \eta^{2} } }}.} \\ \end{array}$$

To avoid circular convolution errors, the $N \times N$ array $\overline{P}_{S} \left( {\xi ,\eta } \right)$ has to be padded by at least $N - 1$ zeros^37,43^. According to convention, the convolution kernel array and the zero-padded pressure field array are of the same size^37,43^. The $N \times N$ subarray at the center of the larger array generated as the output of FFT-based convolution is $\overline{P}_{M - } \left( {x,y} \right)$.

Subsequently, we apply the phase shift due to the discretized metalens to obtain $N \times N$ array $\overline{P}_{M + } \left( {x,y} \right)$, which represents the pressure field at the plane after the metalens. This can be done by performing an element-wise multiplication of the $N \times N$ array $\overline{P}_{M - } \left( {x,y} \right)$ and the $N \times N$ array $\exp \left( {i\overline{\phi }_{discretized} } \right)$. Note that $\overline{\phi }_{discretized}$ is the metalens’ discretized phase profile after the interpolation step in the ultrasonic metalens designing process.

Following this, we use FFT-based convolution to convolve the zero-padded array $\overline{P}_{M + } \left( {x,y} \right)$ with the convolution kernel

22 $$\begin{array}{*{20}c} {\overline{h}_{2} \left( {x,y} \right) = \left( {\frac{1}{{\sqrt {f^{2} + x^{2} + y^{2} } }} + jk} \right)\frac{{f\exp \left( { - jk\sqrt {f^{2} + x^{2} + y^{2} } } \right)}}{{2\pi \left( {f^{2} + x^{2} + y^{2} } \right)}}} \\ \end{array}$$

in order to obtain the $N \times N$ output pressure field array $\overline{P}_{O} \left( {u,v} \right)$.

The convolution kernels in Eqs. (21) and (22) were derived from exact solutions to the Kirchhoff-Helmholtz Integral^37^.

#### Propagation through the SFM

The SFM theoretically applies the transfer function (TF) needed to solve a particular ODE or PDE. To simulate ultrasonic wave propagation through the SFM, we perform element-wise multiplication of the output array of the first UFT block and the transmission coefficient array $T\left( {x,y} \right)$ of the SFM. The result is then the input array for the second UFT block. We subsequently repeat the same process described above for the simulation of wave propagation through a UFT block, which ultimately produces the output $P_{O} \left( {x,y} \right)$ of the proposed ACS.

### Simulation parameters

The process of selecting the most appropriate simulation parameters involves three key considerations. Firstly, convolution requires that the spacing ${\Delta }$ between adjacent metalens unit cells and that between the sampled points of the pressure fields must be the same^37,43^. Furthermore, an appropriate length $L$ for the cross-section of the UFT block should be chosen, keeping in mind that the geometric spread parameter $w$ or $\gamma$ must take on a moderate value so that the significant space and spatial frequency components are within the sampled array bounds (appropriate truncation and bandlimiting). Finally, the focal length $f$ ought to satisfy the condition

23 $$\begin{array}{*{20}c} {f \ge \sqrt {\left[ {\frac{{2\left( {L - {\Delta }} \right){\Delta }}}{\lambda }} \right]^{2} - \left( {L - {\Delta }} \right)^{2} } ,} \\ \end{array}$$

which can be derived by considering the sampling requirements of the convolution kernels’ exponential phase term^36,37,40–44^.

With these considerations in mind, the values of the parameters used in the simulations are presented in Table 2.

**Table 2:** Values of simulation parameters used.

Parameter	Value
${\Delta }$	3 µm
$L$	771 µm
$f_{wave}$	1.7 GHz
$v_{wave}$	5880 m s^-1^
$f$	1.0886 mm

### Supplementary Information

Supplementary Figures.

## Data Availability

The datasets generated during and/or analyzed during the current study are available from the corresponding author on reasonable request.
