# Supplementary material for: Solving ordinary and partial differential equations using an analog computing system based on ultrasonic metasurfaces
Source: Sci Rep. 2023 Aug 18;13:13471. doi: 10.1038/s41598-023-38718-1 (PMC10439223; doi:10.1038/s41598-023-38718-1)
Supplement: Supplementary file 1 — Supplementary Figures. [file 41598_2023_38718_MOESM1_ESM.docx]

**Supplementary Information:**

**Solving ordinary and partial differential equations using an analog computing system based on ultrasonic metasurfaces**

**Robert Frederik Uy**^1,*^ **and Viet Phuong Bui**^2^_­_

^1^Hwa Chong Institution, 661 Bukit Timah Road, Singapore, 269734, Singapore

^2^Institute of High Performance Computing (IHPC), Agency for Science, Technology and Research (A*STAR), 1 Fusionopolis Way, #16-16 Connexis, Singapore, 138632, Republic of Singapore

*Corresponding Author: robertfrederikduy@gmail.com

**Supplementary Figure S1**

| 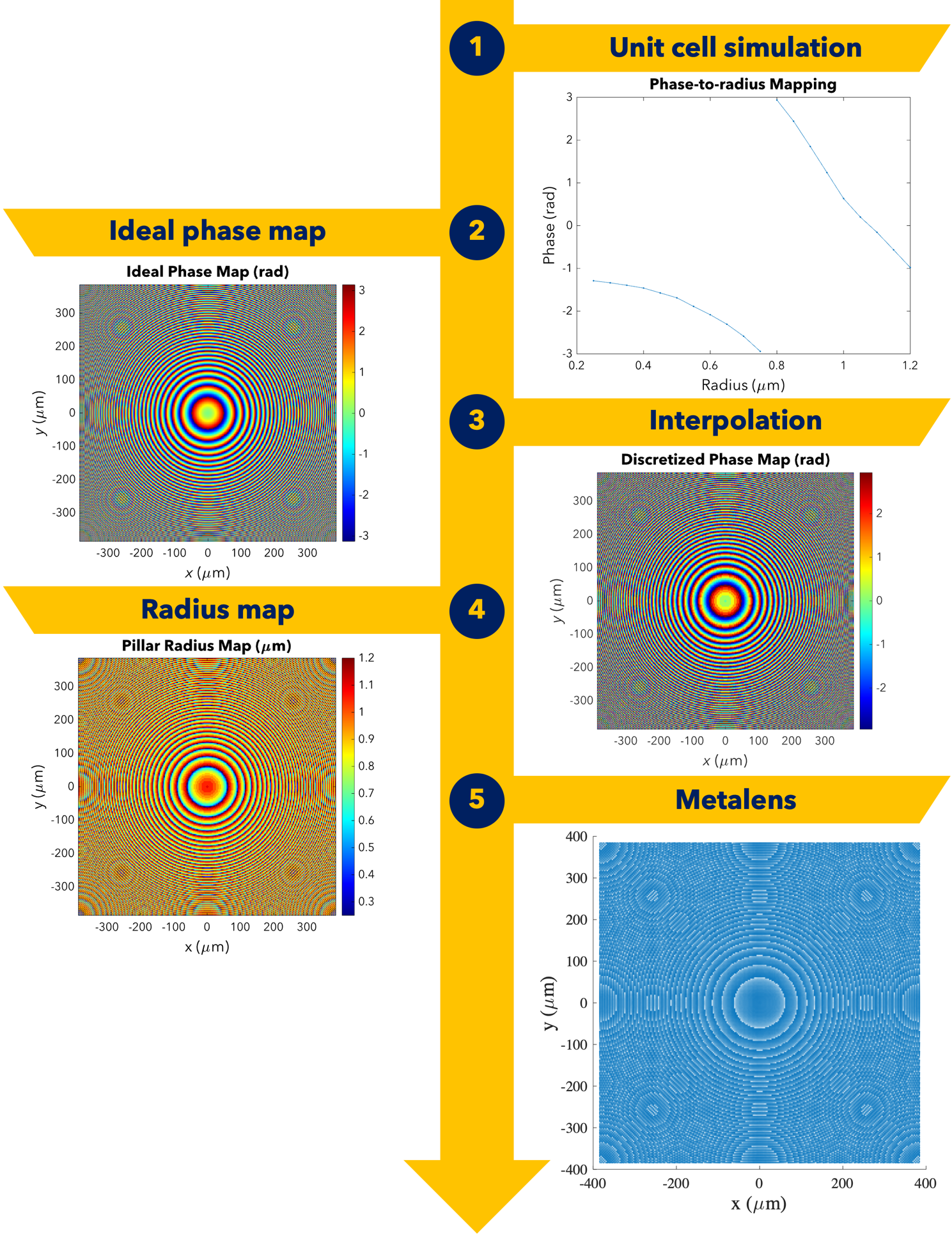 |
| --- |
| **Supplementary Figure S1. Metalens Designing Process.** The flowchart shows the five-step process of designing the ultrasonic metalens. This process yields the full top view of the metalens designed for the proposed ACS. This figure was adapted from Ref. 37. |

**Supplementary Note: Ordinary Differential Equation (ODE) Extreme Cases**

Our simulations of the ACS solving ODEs have demonstrated that accuracy is optimized when the geometric spread parameter $w$ or $\gamma$ is moderate. To support the explanations provided in the main text, we carried out simulations on Sinc and Gaussian functions with extreme values for the geometric spread parameter $w$ or $\gamma$.

Firstly, we consider a Sinc function with parameter $w=6$ (very small). Through our simulations, we have determined the RMSE to be 0.0622. Supplementary Fig. S2 shows the magnitude profiles of the pressure fields at the input plane, the plane before the SFM, the plane after the SFM, and the output plane.

| 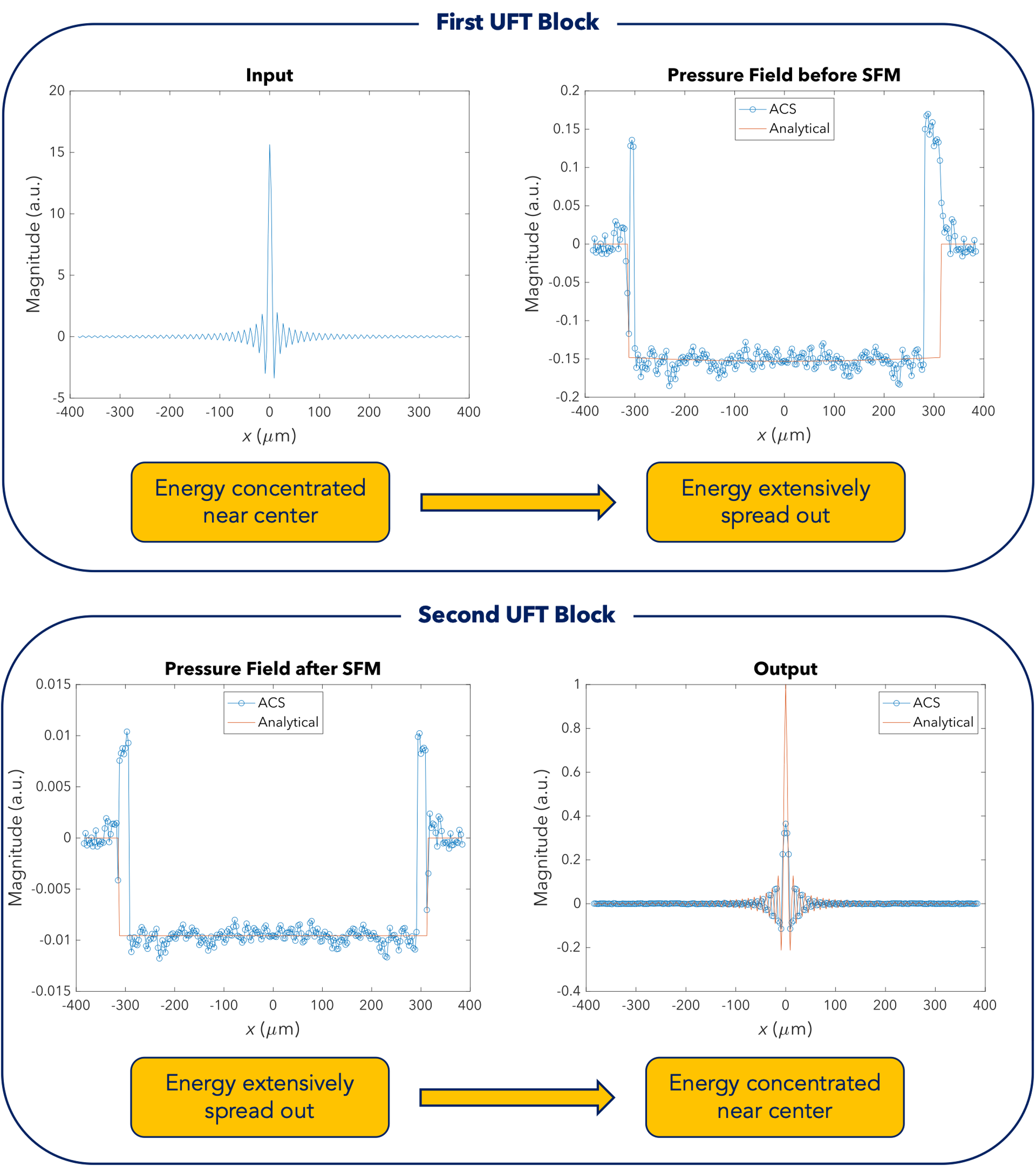 |
| --- |
|  |
| **Supplementary Figure S2. Solving ODEs: Sinc with small** $\boldsymbol{w}$**.** Magnitude profiles of the pressure fields at the input plane, the plane before the SFM, the plane after the SFM, and the output plane. |

Next, we consider a Sinc function with parameter $w=84$ (very large). Through our simulations, we have determined the RMSE to be 0.0542. Supplementary Fig. S3 shows the magnitude profiles of the pressure fields at the input plane, the plane before the SFM, the plane after the SFM, and the output plane.

Notice that there are ripple artifacts in the pressure field before the SFM and that after the SFM due to truncation of the input function.

| 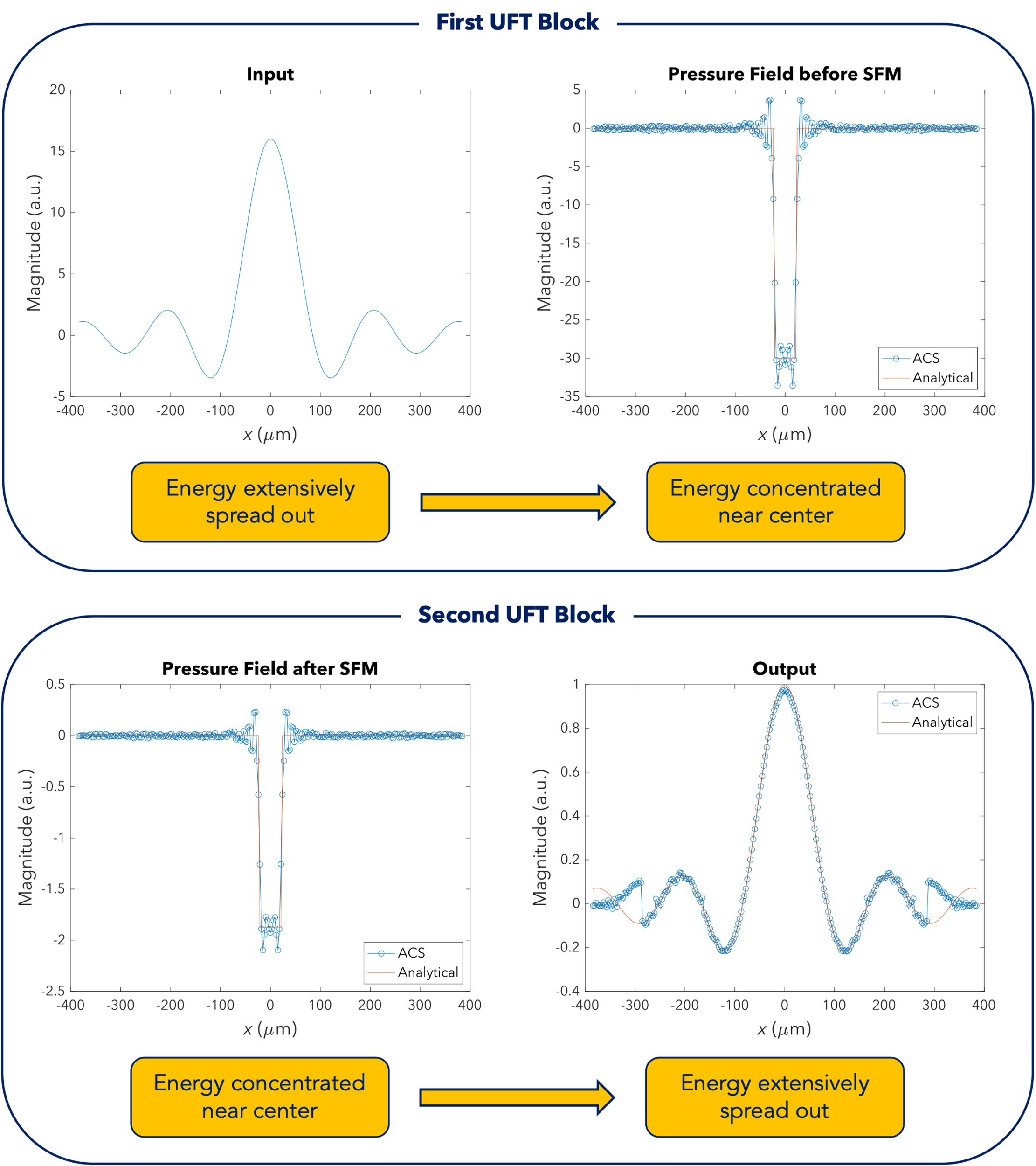 |
| --- |
|  |
| **Supplementary Figure S3. Solving ODEs: Sinc with large** $\boldsymbol{w}$**.** Magnitude profiles of the pressure fields at the input plane, the plane before the SFM, the plane after the SFM, and the output plane. |

Next, we consider a Gaussian function with parameter $\gamma=4$ (very small). Through our simulations, we have determined the RMSE to be 0.0529. Supplementary Fig. S4 shows the magnitude profiles of the pressure fields at the input plane, the plane before the SFM, the plane after the SFM, and the output plane.

| 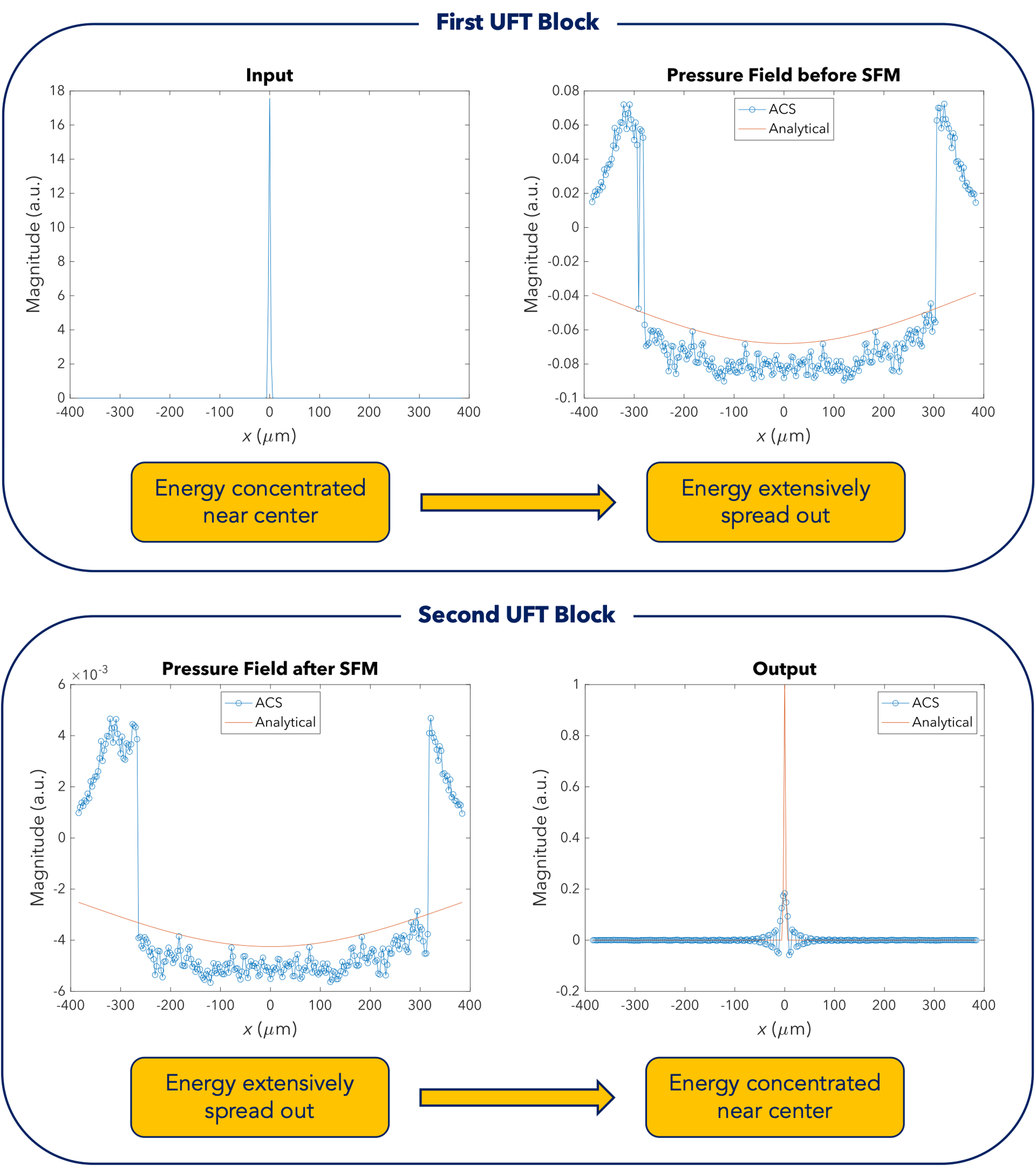 |
| --- |
|  |
| **Supplementary Figure S4. Solving ODEs: Gaussian with small** $\boldsymbol{\gamma}$**.** Magnitude profiles of the pressure fields at the input plane, the plane before the SFM, the plane after the SFM, and the output plane. |

Finally, we consider a Gaussian function with parameter $\gamma=300$ (very large). Through our simulations, we have determined the RMSE to be 0.0399. Supplementary Fig. S5 shows the magnitude profiles of the pressure fields at the input plane, the plane before the SFM, the plane after the SFM, and the output plane.

| 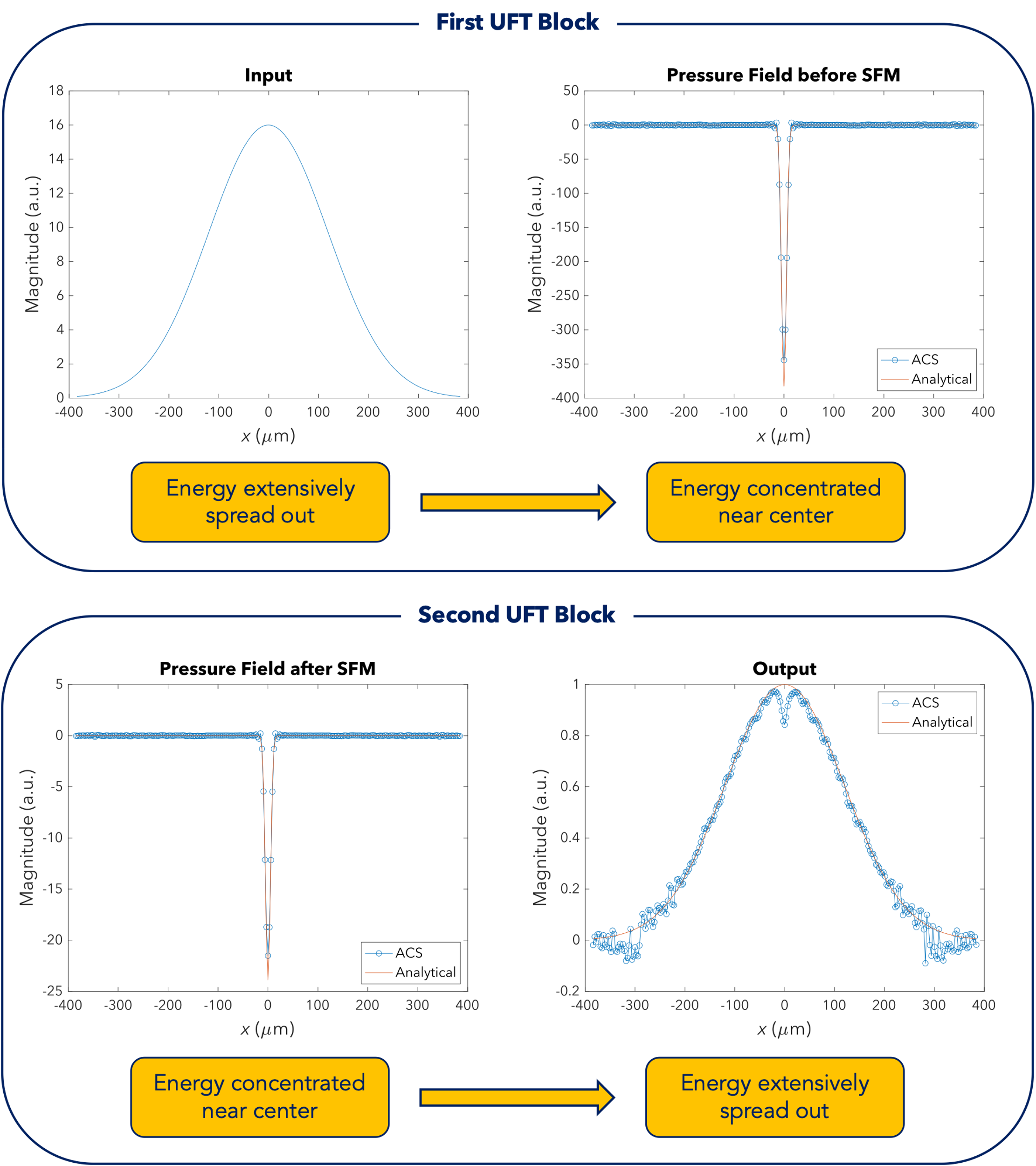 |
| --- |
|  |
| **Supplementary Figure S5. Solving ODEs: Gaussian with large** $\boldsymbol{\gamma}$**.** Magnitude profiles of the pressure fields at the input plane, the plane before the SFM, the plane after the SFM, and the output plane. |

**Supplementary Note: Partial Differential Equation (PDE) Extreme Cases**

Our simulations of the ACS solving PDEs have demonstrated that accuracy is optimized when the geometric spread parameter $w$ or $\gamma$ is moderate. To support the explanations provided in the main text, we carried out simulations on Sinc and Gaussian functions with extreme values for the geometric spread parameter $w$ or $\gamma$.

Firstly, we consider a Sinc function with parameter $w=6$ (very small). Through our simulations, we have determined the RMSE to be 0.0537. Supplementary Fig. S6 shows the magnitude profiles of the pressure fields at the input plane, the plane before the SFM, the plane after the SFM, and the output plane.

| 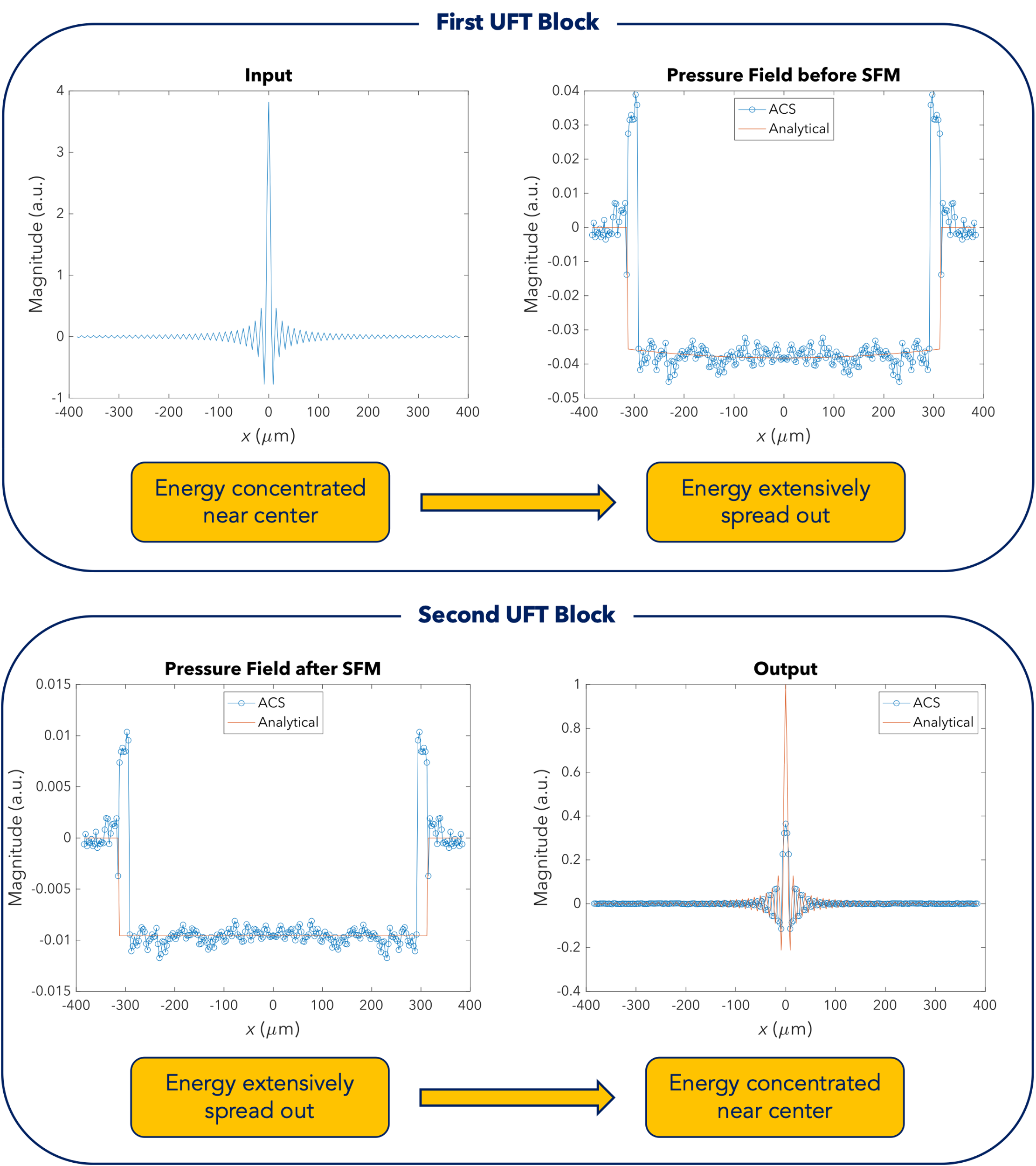 |
| --- |
|  |
| **Supplementary Figure S6. Solving PDEs: Sinc with small** $\boldsymbol{w}$**.** Magnitude profiles of the pressure fields at the input plane, the plane before the SFM, the plane after the SFM, and the output plane. |

Next, we consider a Sinc function with parameter $w=90$ (very large). Through our simulations, we have determined the RMSE to be 0.0515. Supplementary Fig. S7 shows the magnitude profiles of the pressure fields at the input plane, the plane before the SFM, the plane after the SFM, and the output plane.

Notice that there are ripple artifacts in the pressure field before the SFM and that after the SFM due to truncation of the input function.

| 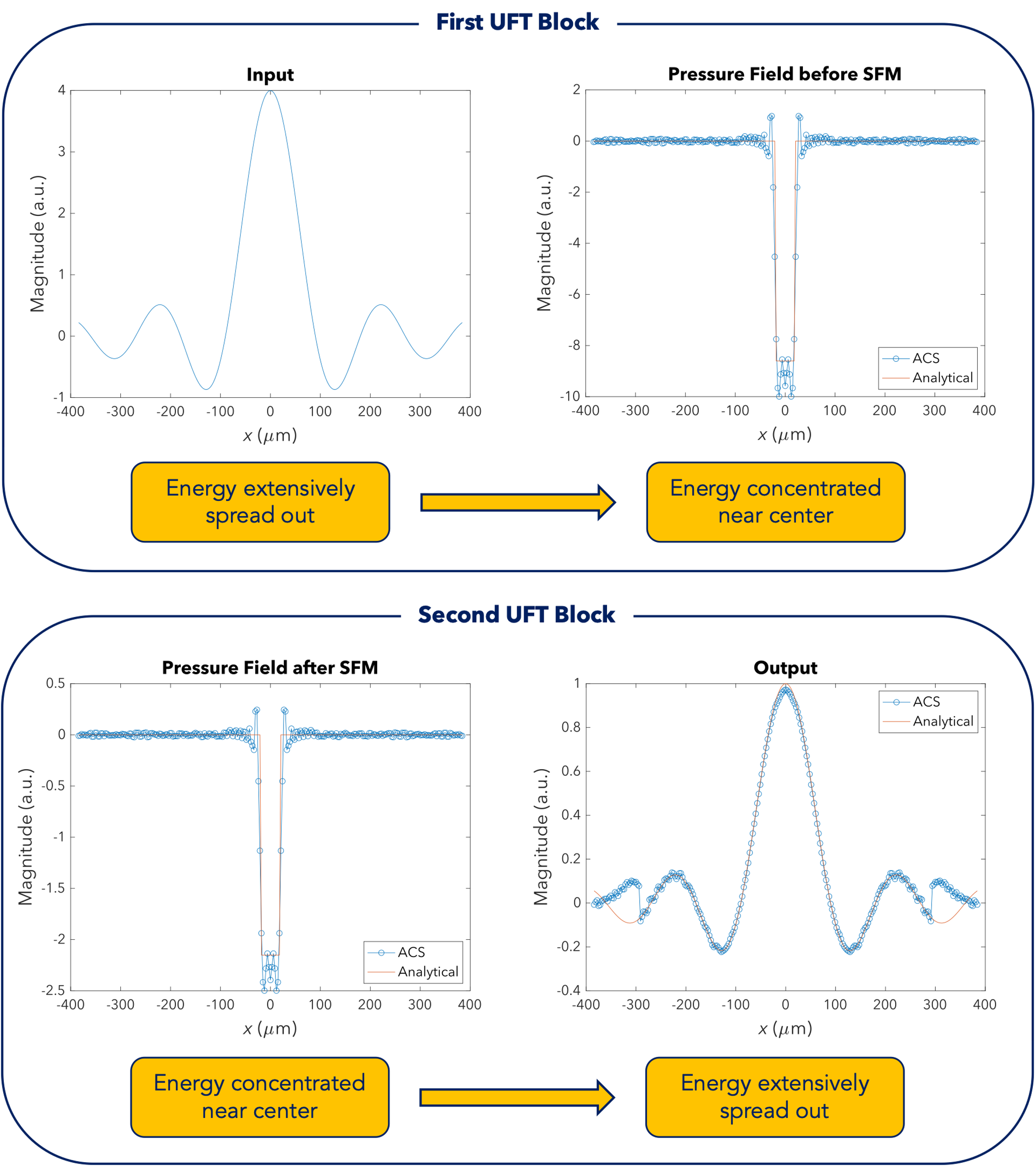 |
| --- |
|  |
| **Supplementary Figure S7. Solving PDEs: Sinc with large** $\boldsymbol{w}$**.** Magnitude profiles of the pressure fields at the input plane, the plane before the SFM, the plane after the SFM, and the output plane. |

Next, we consider a Gaussian function with parameter $\gamma=6$ (very small). Through our simulations, we have determined the RMSE to be 0.0449. Supplementary Fig. S8 shows the magnitude profiles of the pressure fields at the input plane, the plane before the SFM, the plane after the SFM, and the output plane.

| 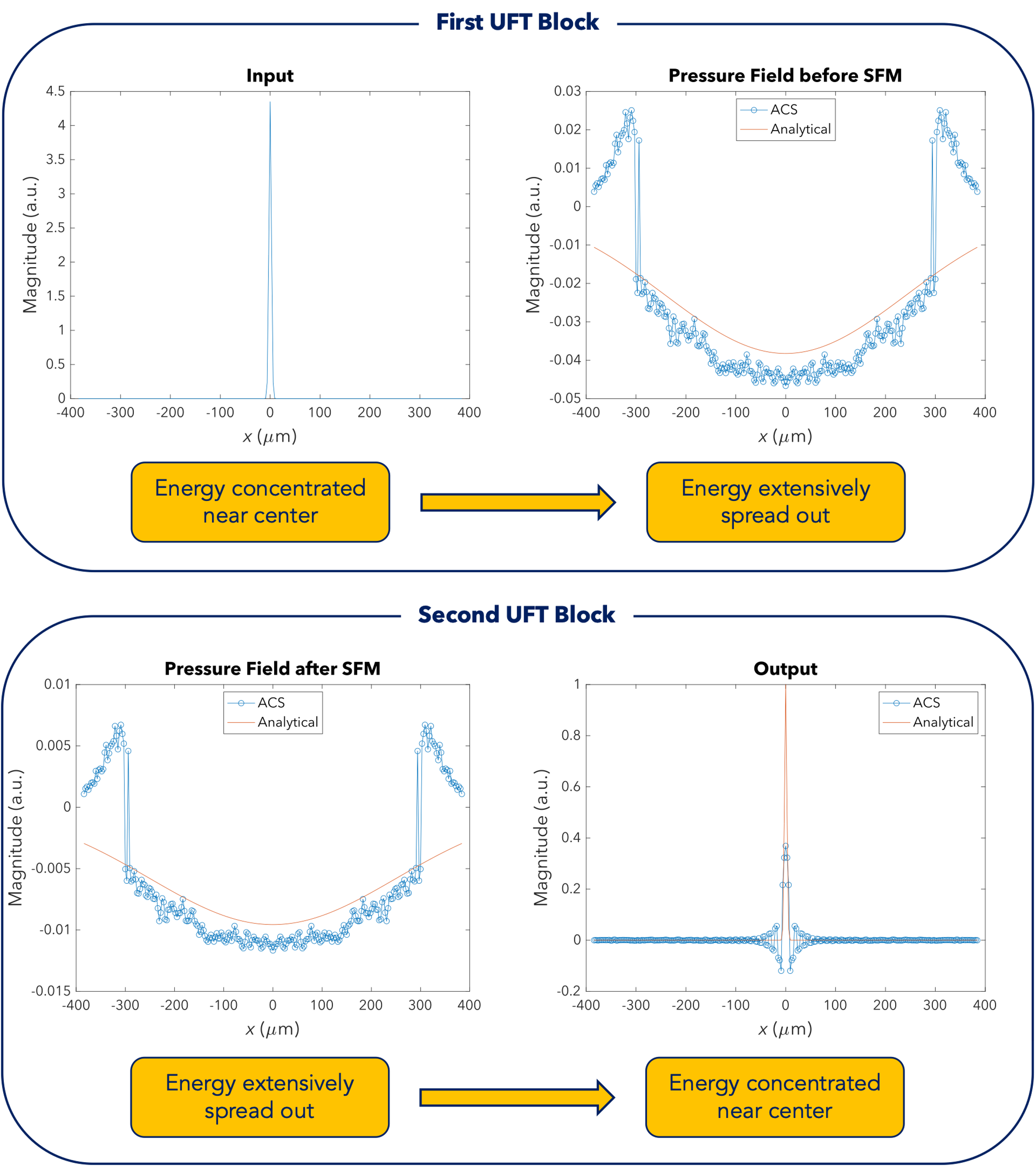 |
| --- |
|  |
| **Supplementary Figure S8. Solving PDEs: Gaussian with small** $\boldsymbol{\gamma}$**.** Magnitude profiles of the pressure fields at the input plane, the plane before the SFM, the plane after the SFM, and the output plane. |

Finally, we consider a Gaussian function with parameter $\gamma=300$ (very large). Through our simulations, we have determined the RMSE to be 0.0276. Supplementary Fig. S9 shows the magnitude profiles of the pressure fields at the input plane, the plane before the SFM, the plane after the SFM, and the output plane.

| 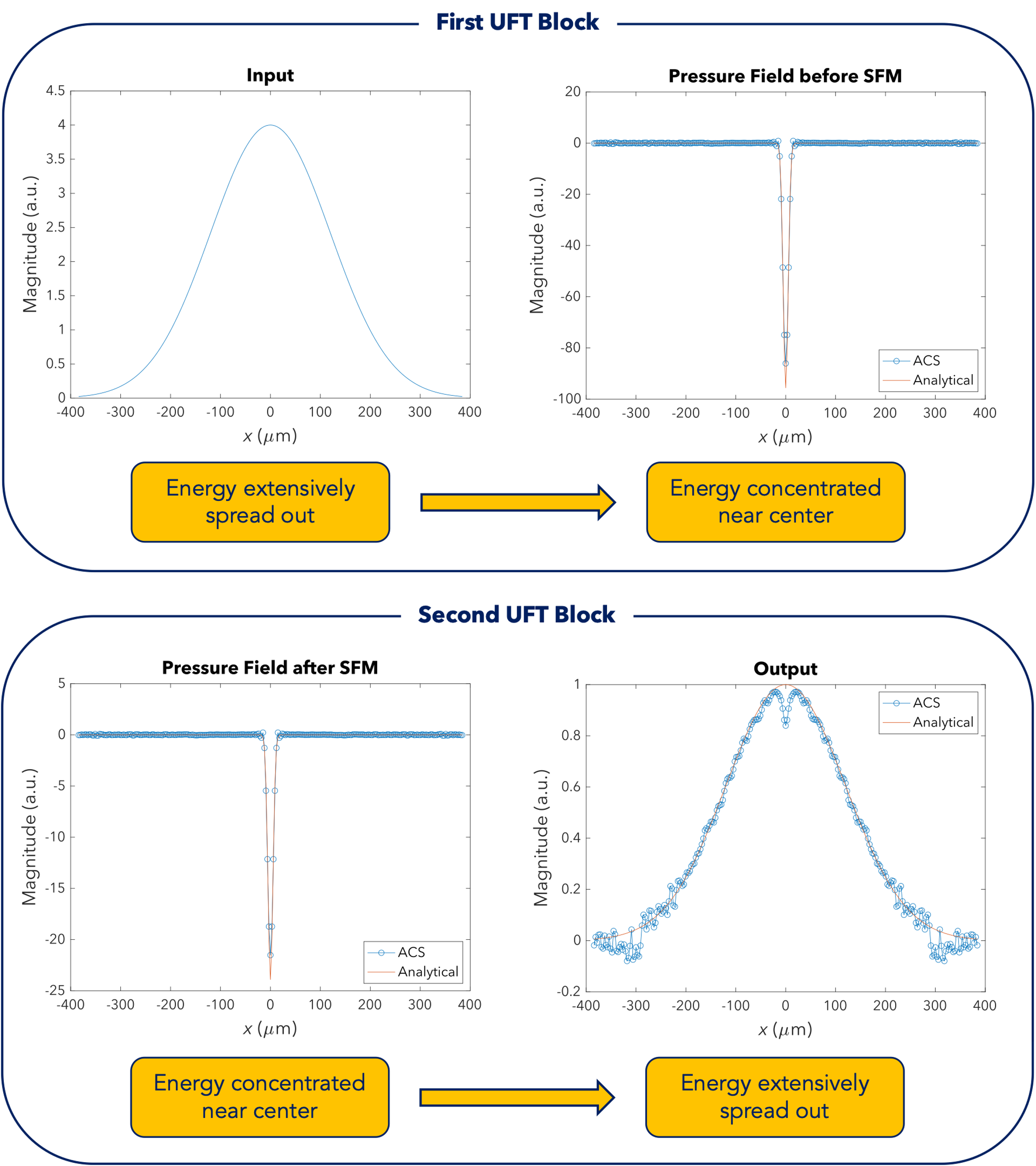 |
| --- |
|  |
| **Supplementary Figure S9. Solving PDEs: Gaussian with large** $\boldsymbol{\gamma}$**.** Magnitude profiles of the pressure fields at the input plane, the plane before the SFM, the plane after the SFM, and the output plane. |
